# Similar Clinical and Surgical Outcomes Achieved with Early Compared to Late Anti-TNF Induction in Mild-to-Moderate Ulcerative Colitis: A Retrospective Cohort Study

**DOI:** 10.1155/2016/2079582

**Published:** 2016-07-11

**Authors:** Christopher Ma, Candace L. Beilman, Vivian W. Huang, Darryl K. Fedorak, Karen Wong, Karen I. Kroeker, Levinus A. Dieleman, Brendan P. Halloran, Richard N. Fedorak

**Affiliations:** Division of Gastroenterology, University of Alberta, Edmonton, AB, Canada T6G 2X8

## Abstract

*Background.* Biologic agents targeting tumor necrosis factor alpha are effective in the management of ulcerative colitis (UC), but their use is often postponed until after failure of other treatment modalities.* Objectives.* We aim to determine if earlier treatment with infliximab or adalimumab alters clinical and surgical outcomes in UC patients.* Methods.* A retrospective cohort study was conducted evaluating UC outpatients treated with infliximab or adalimumab from 2003 to 2014. Patients were stratified by time to first anti-TNF exposure; early initiation was defined as starting treatment within three years of diagnosis. Primary outcomes were colectomy, UC-related hospitalization, and clinical secondary loss of response. Kaplan-Meier analysis was used to assess time to the primary outcomes.* Results.* 115 patients were included (78 infliximab, 37 adalimumab). Median follow-up was 175.6 weeks (IQR 72.4–228.4 weeks). Fifty-seven (49.6%) patients received early anti-TNF therapy; median time to treatment in this group was 38.1 (23.3–91.0) weeks compared to 414.0 (254.0–561.3) weeks in the late initiator cohort (*p* < 0.0001). Patients treated with early anti-TNF therapy had more severe endoscopic disease at induction (mean Mayo endoscopy subscore 2.46 (SD ± 0.66) versus 1.86 (±0.67), *p* < 0.001) and trended towards increased risk of colectomy (17.5% versus 8.6%, *p* = 0.16) and UC-related hospitalization (43.9% versus 27.6%, *p* = 0.07). In multivariate regression analysis, early anti-TNF induction was not associated with colectomy (HR 2.02 [95% CI: 0.57–7.20]), hospitalization (HR 1.66 [0.84–3.30]), or secondary loss of response (HR 0.86 [0.52–1.42]).* Conclusions.* Anti-TNF therapy is initiated earlier in patients with severe UC but earlier treatment does not prevent hospitalization, colectomy, or secondary loss of response.

## 1. Introduction

Ulcerative colitis (UC) is a chronic relapsing and remitting condition causing large intestine inflammation due to immune dysregulation. UC carries a substantial burden of associated health care costs and patient morbidity: over 100,000 Canadians are living with UC and total direct medical costs associated with inflammatory bowel disease (IBD) were estimated at $1.2 billion in 2012, largely driven by the costs of hospitalization and surgery [1]. Ten years after diagnosis, approximately 10% of UC patients will require colectomy for management of medically refractory disease or disease-related complications [2]. Although colectomy remains an important component of UC management, it is not without risk as nearly 30% of patients undergoing colectomy experience a postoperative complication [3].

The medical armamentarium for treatment of UC has dramatically changed over the past decade with the introduction of biologic agents targeting tumor necrosis factor alpha (TNF-*α*), including infliximab and adalimumab. Multiple randomized controlled trials have demonstrated the efficacy of both agents for induction and maintenance of clinical response and remission in patients with moderate-to-severe UC failing conventional therapy with corticosteroids and immunomodulators [4, 5]. These agents have also played a key role in facilitating the paradigm shift towards objective UC treatment outcome measures: aggressive anti-TNF therapy targeting mucosal healing has been demonstrated to decrease the risk of colectomy and corticosteroid dependence [6]. Furthermore, the use of these agents may be starting to change the natural history of UC: in a large cohort study including 481 UC patients undergoing colectomy, Reich et al. demonstrated an average annual decrease in colectomy rate by −16.1% [95% CI: −21.3% to −10.5%] from 2005–2011, corresponding to a significant increase in use of anti-TNF agents [7]. Similarly, in a large population-based time trend analysis from 1997 to 2009 using administrative data, Kaplan et al. report a 13% per year adjusted risk reduction in odds of colectomy (OR 0.87 [95% CI: 0.83–0.902] and average annual percent change of –7.4% [95% CI: −10.8%, −3.9%]) for elective colectomies, corresponding to increasing use of immunomodulators and infliximab [8].

The recent Toronto Consensus clinical practice guidelines now strongly recommend the use of anti-TNF therapy for induction and maintenance therapy in UC patients failing to respond to thiopurines or corticosteroids [9]. However, there is still reluctance to use these agents early in the disease course for UC, potentiated by misconceptions regarding UC- and colectomy-related morbidity and efficacy of anti-TNF therapy for UC as compared to Crohn's disease (CD) [10]. For CD, there is evidence to suggest that earlier initiation of anti-TNF therapy modulates long-term clinical outcomes: in post hoc analysis of the CHARM and ADHERE trials, Schreiber et al. reported a 15% improvement in week 56 remission rates for patients starting adalimumab within the first two years of diagnosis compared to initiation after five years of disease (43% versus 28%, *p* < 0.001) [11] and data from our centre demonstrates a significant reduction in surgical bowel resection (30.7% versus 5.7%, *p* < 0.001) in CD patients started on early anti-TNF therapy [12].

However, results from CD studies may not be generalizable to the UC population and no previous studies have directly evaluated the effect of earlier introduction of anti-TNF therapy on the natural history of UC. In this study, we assessed the effect of early initiation of infliximab or adalimumab, within three years of diagnosis, on the rate of colectomy, UC-related hospitalization, and secondary clinical loss of response during maintenance therapy.

## 2. Materials and Methods

### 2.1. Study Design, Setting, and Data Source

This retrospective cohort study was performed using data collected from UC outpatients receiving infliximab or adalimumab between January 2003 and December 2014, at the University of Alberta Inflammatory Bowel Disease Consultation and Research Clinic, Edmonton, Alberta, Canada. Patients were identified from the Division of Gastroenterology IBD Electronic Database. Electronic records were available and reviewed up to December 31, 2014.

### 2.2. Patient Population

Patients were eligible for inclusion if they met the following criteria: (1) confirmed UC with an established date of diagnosis by either endoscopy or histology (for patients diagnosed prior to the introduction of the electronic medical record in 2002, date of diagnosis was confirmed using the paper medical chart); (2) achieved primary response within 12 to 14 weeks of induction therapy (decrease in partial Mayo score of >2 points) with infliximab 5 mg/kg at weeks 0, 2, and 6 or adalimumab 160 mg at week 0 and 80 mg at week 2; and (3) started on maintenance anti-TNF therapy after primary induction response of infliximab 5 mg/kg every 8 weeks or adalimumab 40 mg every other week. Minimum follow-up duration was 16 weeks. Patients were excluded if they were primary nonresponders to anti-TNF induction therapy, received induction therapy while being hospitalized as an inpatient, or had a previous history of colectomy prior to anti-TNF induction. We specifically excluded primary nonresponders and inpatients to minimize the heterogeneity of our cohort and limit the effect of disease severity on selection bias towards early anti-TNF initiators. Initial choice of anti-TNF agent (i.e., infliximab versus adalimumab) was at the discretion of the patient and their attending gastroenterologist.

Patients were subsequently stratified by time to their first dose of induction of anti-TNF agent: early initiation of anti-TNF therapy was defined as starting infliximab or adalimumab within three years of diagnosis. This time interval was determined in accordance with previous studies, including analysis of randomized controlled trials, to facilitate comparison with the existing literature [13].

### 2.3. Data Collection

Data was extracted by authors Christopher Ma and Darryl K. Fedorak from two sources using a standardized case report form: (1) physician office-based electronic files (including all clinic follow-up notes, nursing and direct patient correspondence, outpatient prescriptions, and consultation letters) and (2) region-wide electronic health care database (including all inpatient and outpatient laboratory investigations, diagnostic imaging, histology and pathology reports, hospital admission and discharge summaries, and operative reports including endoscopy reports).

Baseline patient data collected included gender, age, date of diagnosis, smoking status, previous treatments for UC, concurrent medications at anti-TNF induction, disease extent per Montreal Classification for UC [14], endoscopic Mayo subscore at diagnosis, and subjective partial Mayo score at anti-TNF induction [15]. To evaluate potential temporal relationships in outcomes based on year of therapy and to adjust for changes in treatment paradigms during the study inclusion period, we divided the cohort into patients receiving induction therapy within 2003–2008 and 2009–2014.

### 2.4. Outcomes

The primary objective of this study was to determine if early initiation of anti-TNF therapy within three years of diagnosis in UC outpatients affected (1) the need for colectomy, (2) the need for UC-related hospitalization, or (3) clinical secondary loss of response requiring dose escalation of infliximab or adalimumab during maintenance therapy.

The primary surgical outcome for this study was colectomy, including proctocolectomy or total abdominal colectomy, with or without pouch or stoma formation. We included colectomies performed for any indication including medically refractory disease, disease-related complications (e.g., toxic megacolon), or colorectal dysplasia or cancer. The primary surgical outcome was further categorized by surgical urgency (elective versus emergent surgery).

Primary clinical outcomes were UC-related hospitalization and secondary loss of response. Hospitalizations were identified from the region-wide electronic health care database visit log and UC-related hospitalizations were defined by hospitalization for primary diagnosis of UC-related flare or complications related to UC treatment. Admissions to hospital for other comorbid medical conditions were excluded. Secondary loss of response requiring dose escalation of anti-TNF therapy as determined by the attending gastroenterologist based on a structured institutional protocol incorporating patient reported symptoms by partial Mayo score (increase in score >2), elevated inflammatory markers (CRP >8.0 mg/L, fecal calprotectin >150 *μ*g/g), and endoscopic evidence of disease activity, with exclusion of enteric infections including* C. difficile*. Dose escalation was defined as an increase in administered anti-TNF dose or a shortened interval of administration. For infliximab, dose escalation consisted of increasing the dose to 10 mg/kg per infusion or increasing the frequency of infusions to less than every eight weeks. A dose increase due to increased patient weight was not considered therapeutic escalation. For adalimumab, escalation consisted of increasing the dose to 80 mg per injection or increasing the frequency to weekly injections. Routine measurement of anti-TNF drug levels or anti-drug antibodies was not consistently available during the study period for analysis, so dose escalation was based on institutional protocol rather than therapeutic drug monitoring.

Total follow-up time was determined from the start of therapy to the last date of anti-TNF administration. Patients who maintained clinical response without need for dose escalation or colectomy from induction to the end of the study period were considered censored cases.

### 2.5. Statistical Methods

For continuous variables, mean and standard deviation (for normally distributed) and median and interquartile range (for nonnormally distributed variables) were calculated. Normality of continuous variables was assessed using the Shapiro-Wilks method. Patient variables in the early initiator and late initiator groups were compared using the student* t-*test and Chi-squared test. The Kaplan-Meier method was used to assess probability of colectomy, hospitalization, and clinical secondary loss of response during maintenance infliximab and adalimumab therapy over time. A log-rank test for equality of survivor functions was performed, where a *p* value < 0.05 was considered the level of statistical significance.

As there is no well-validated definition of early UC and to adjust for confounding variables that may affect the primary clinical and surgical outcomes, we performed a univariate and multivariate regression analysis to evaluate clinical predictors of colectomy, hospitalization, and loss of response. A Cox proportional hazards regression model (enter method) was used and stepwise purposeful selection of variables with a *p* value <0.2 in univariate analysis was included in the multivariate regression. Confounder variables selected for univariate analysis included gender, age, disease extent by Montreal Classification, early versus late anti-TNF induction, year of anti-TNF induction, C-reactive protein, partial Mayo score, endoscopic Mayo sub-score at diagnosis and at anti-TNF induction, and concurrent steroids, immunomodulators, and 5-ASA therapy.

Statistical analysis was performed with SPSS 22.0 statistical software (Armonk, NY: IBM Corporation).

## 3. Results

### 3.1. Baseline Patient Demographics

Patient demographics are summarized in Table 1. One hundred fifteen UC patients met study inclusion criteria. Seventy-eight patients (67.8%) were treated with infliximab and 37/115 (32.2%) were treated with adalimumab. Anti-TNF therapy was initiated within the first three years of diagnosis in 57 patients (49.6%). Median time to first induction dose of infliximab or adalimumab in this early initiator cohort was 38.1 weeks (IQR 23.3–91.0 weeks); in comparison, median time to induction in the late initiator group was 414.0 weeks (IQR 254.0–561.3 weeks) (*p* < 0.0001). Eighteen patients (15.7%) received their first anti-TNF induction dose more than ten years after diagnosis; 49 patients (42.6%) received anti-TNF induction prior to 2009.

Patients in the early initiator cohort had more active disease. Mean endoscopic Mayo subscore at anti-TNF induction was 2.46 (±0.66) for early initiators compared to 1.86 (±0.67) for late initiators (*p* < 0.001). Median C-reactive protein was also significantly higher in early initiators (35.7 mg/L [IQR 9.2–80.0] versus 6.4 mg/L [2.0–13.6], *p* < 0.001). Early initiators trended towards having higher partial Mayo score at induction (6.38 versus 5.67, *p* = 0.08) and were more likely to require concurrent corticosteroids at anti-TNF induction (70.2% versus 55.2%, *p* = 0.10).

Mean total follow-up duration was 153.4 (±84.3) weeks for early and 168.3 (±106.4) weeks for late anti-TNF initiators (*p* = 0.41). Total follow-up in the cohort included 355 cumulative patient-years of anti-TNF exposure.

### 3.2. Surgical Outcomes: Colectomy

**Figure 1:**
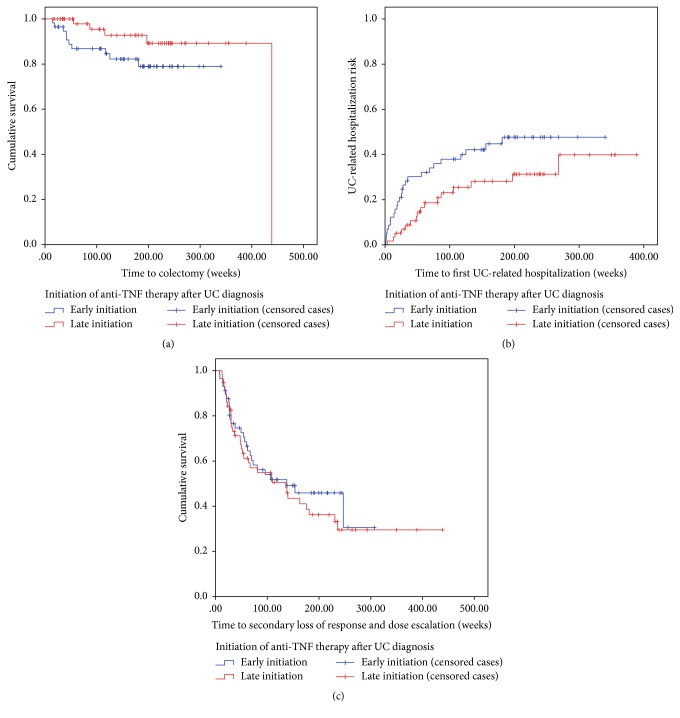
Kaplan-Meier survival curves demonstrating trend towards increased colectomy (a, *p* = 0.06) and UC-related hospitalization (b, *p* = 0.02) rate during maintenance infliximab or adalimumab for 57 ulcerative colitis patients starting anti-TNF therapy within three years of diagnosis (blue) compared to 57 patients starting anti-TNF therapy more than three years after diagnosis (red). There is no difference in secondary loss of response requiring dose escalation between early and late anti-TNF initiators (c, *p* = 0.70). Hashed lines indicate censored cases (which did not meet primary outcome to last follow-up).

Fifteen UC patients (13.0%) required colectomy during maintenance anti-TNF therapy (Table 2). Quantitatively, more patients in the early initiator cohort required colectomy (10/57 [17.5%] versus 5/58 [8.6%]) but this was not statistically significant (*p* = 0.16). Similarly, early initiators trended towards earlier colectomy (median time to surgery after anti-TNF induction 49.1 weeks versus 119.0 weeks, *p* = 0.61), and this was also observed on Kaplan-Meier analysis (Figure 1(a), *p* = 0.06). Colectomy rate was higher in the early initiator cohort (6.0 colectomies per 100 patient-years of treatment) compared to late initiators (2.7 colectomies per 100 patient-years of treatment) but this was not statistically significant (*p* = 0.13). The majority of patients required colectomy for medically refractory disease.

### 3.3. Clinical Outcomes: Hospitalization and Secondary Loss of Response

Forty-one patients (35.7%) required hospital admission for UC during the follow-up period, generating 71 hospitalizations (Table 2). Early initiation of anti-TNF therapy was associated with a trend towards increased hospitalization (43.9% versus 27.6%, *p* = 0.07) and earlier hospitalization after anti-TNF therapy (Figure 1(b), *p* = 0.02). Median time to hospitalization in late initiators was approximately twice that of early initiators (52.5 weeks versus 25.6 weeks, *p* = 0.08) and rate of UC-related hospitalization was significantly higher in early initiators (26.8 versus 13.9 hospitalizations per 100 patient-years of treatment, *p* = 0.01).

Sixty-two patients (53.9%) experienced a secondary loss of response requiring anti-TNF dose escalation during maintenance therapy (Table 2). A similar proportion of late and early anti-TNF initiators developed secondary loss of response (58.6% versus 49.1%, *p* = 0.31).

### 3.4. Univariate and Multivariate Cox Proportional Hazards Model

Hazard ratios (HR) in univariate and multivariate Cox proportional hazards models are summarized in Table 3. In univariate analysis, patients receiving early anti-TNF therapy trended towards an increased risk of UC-related hospitalizations (HR 1.83 [0.98–3.43]) and colectomy (HR 2.56 [0.80–8.19]) but this was not statistically significant and the trend was further reduced after adjustment in multivariate analysis for endoscopic Mayo subscore at anti-TNF induction and year of anti-TNF induction. Induction therapy after 2009 was identified as a risk factor for clinical secondary loss of response in multivariate analysis after adjusting for C-reactive protein, Mayo endoscopic activity subscore, and concurrent steroid use at anti-TNF induction although the effect size is small (HR 1.95 [1.05–3.61]).

Choice of anti-TNF agent, gender, age, disease extent by Montreal Classification, Mayo endoscopic subscore at diagnosis, and concurrent use of immunomodulators or 5-ASA agents was not predictive of hospitalization, colectomy, or clinical secondary loss of response in univariate analysis (*p* value >0.20).

## 4. Discussion

The management of moderate-to-severe UC has undergone dramatic changes in the past decade. Previous “step-up” therapy paradigms reserving anti-TNF agents until failure of conventional treatments and treatment targeting symptomatic control have been supplanted by an increasing willingness to use aggressive medical therapy, including biologic agents, early in the disease course to achieve mucosal healing and induce deep remission [9]. In CD, there is increasing evidence that earlier initiation of anti-TNF therapy improves clinical and surgical outcomes [11]. However, the literature evaluating the effect of early introduction of infliximab or adalimumab on the natural history of UC is still needed. Here, we present a retrospective evaluation of 115 UC outpatients on maintenance anti-TNF therapy and do not demonstrate a significant difference in colectomy, UC-related hospitalizations, or clinical secondary loss of response with earlier anti-TNF induction within three years of diagnosis.

Long-term follow-up from randomized controlled trials demonstrates the efficacy of infliximab for reducing the risk of colectomy in UC compared to placebo. In analysis of 728 UC patients in the landmark Active Ulcerative Colitis (ACT) trials, Sandborn et al. report a cumulative absolute risk reduction for colectomy of 7% (17% versus 10%, *p* = 0.02) at 54 weeks with infliximab compared to placebo [13]. Interestingly though, there was no difference in cumulative colectomy incidence when patients treated with 5 mg/kg infliximab were compared to the placebo group (12% versus 17%, *p* = 0.17). Furthermore, in analysis of the Ulcerative Colitis Long-Term Remission and Maintenance with Adalimumab [ULTRA 1 and ULTRA 2] trials, Feagan et al. also failed to demonstrate a significant decrease in week 8 or week 52 colectomy rates in patients treated with adalimumab compared to placebo [16] despite evidence that adalimumab induces and maintains clinical remission and achieves mucosal healing [17, 18]. One potential explanation for this apparent incongruity is that the studies were underpowered to detect a statistically significant difference in colectomy rates owing to low baseline colectomy incidence (<5% in the ULTRA trials, 9% in the ACT trials).

In their multivariate Cox proportional hazards model, Feagan et al. found disease duration >3 years protective against colectomy (HR 0.36 [95% CI 0.23–0.57], *p* < 0.001) [16] and other authors have similarly reported in open-label cohorts that short disease duration is a predictor of surgery [8, 19, 20]. In our study, we found that when anti-TNF therapy was instituted within the first three years after diagnosis, no differences in colectomy outcomes between early and late initiators were appreciable and this is confirmed on multivariate regression analysis. Mandel et al. came to similar conclusions in a smaller Hungarian cohort of 42 UC patients, with no demonstrable differences in hospitalization or colectomy rate with time to anti-TNF exposure [21]. This is in contrast to evidence from studies in CD, where early anti-TNF induction improves clinical response and reduces the risk for surgical resection [11, 22].

There are multiple hypotheses that may explain this discrepancy. Firstly, patients starting early anti-TNF therapy for UC are more likely to have severe disease. Indeed, we observed substantial differences in symptomatic and serologic inflammatory disease severity between early and late initiators of anti-TNF therapy. Even compared to patients enrolled in the ACT and ULTRA RCTs, our early anti-TNF initiators had a higher burden of inflammation (median CRP 35.7 mg/L versus <10 mg/L). Patients with severe UC are at risk for poor anti-TNF primary response due to accelerated drug clearance, monoclonal antibody catabolism, and immunoglobulin excretion secondary to colonic inflammation [23, 24]. This cohort is less likely to achieve deep remission with induction therapy and subsequently is at higher secondary loss of response, hospitalization, and colectomy [18, 25, 26].

Secondly, patients requiring early anti-TNF therapy were more likely to have severe endoscopic disease. A key strength of this study is that we incorporated objective endoscopic disease activity in multivariate regression models for risk of colectomy and hospitalization. Endoscopic disease severity is a powerful predictor of colectomy and in our cohort, early initiators had higher mean endoscopic Mayo scores at anti-TNF induction (2.46 versus 1.86, *p* < 0.001) compared to late initiators. In a multicentre Italian study, Monterubbianesi et al. found the presence of severe endoscopic lesions defined by deep ulcerations or spontaneous bleeding was associated with increased risk for early colectomy at three months (RR 5.13 [95% CI 1.55–16.96]) and late colectomy risk at 12 months (RR 7.0 [95% CI 1.09–44.7]) [27]. Indeed, when adjusted for endoscopic disease activity, time to anti-TNF therapy did not predict colectomy or hospitalization. While there is strong evidence that infliximab should be used early as rescue therapy for severe or fulminant UC [28], our study suggests that, conversely, other therapeutic options could be optimized for patients with mild-to-moderate disease.

Thirdly, when comparing CD and UC, pathophysiologic mechanisms may contribute to the variance in surgical outcomes with timing of anti-TNF induction. In CD, it is hypothesized that early control of inflammatory disease burden with aggressive medical therapy curtails the development of mechanical complications such as fibrostenotic strictures and penetrating disease requiring surgical management [22]. In contrast, patients with UC have predominantly mucosal rather than transmural inflammation and fibrostenotic disease is rare. Patients with UC may require colectomy for management of colorectal cancer (CRC). Whether anti-TNF therapy modulates this risk remains to be seen as the development of CRC, and consequently the duration of follow-up required to determine changes in dysplasia incidence is lengthy [29]. Additionally, because colectomy is a relatively rare occurrence in UC, a large cohort is required to determine statistically significant changes in colectomy rate.

Differences between the early and late initiator cohorts are challenging to adjust for in a retrospective study design but it would also be difficult to evaluate this in a truly randomized, prospective, blinded fashion. As such, we attempted to adjust for multiple confounders, including serologic, clinical, and endoscopic measures of disease activity. We also specifically restricted the inclusion criteria to UC outpatient primary responders to avoid the confounding effect of acute fulminant UC. This subset of patients has extremely aggressive disease, may have received anti-TNF therapy as a rescue indication, and are at high risk for colectomy; thus, inclusion would have further biased the results against early anti-TNF therapy. Even after taking these covariates into consideration, there may be residual selection bias against early anti-TNF initiators. Thus, we recognize the contrasting argument, that in fact anti-TNF therapy is effective in equalizing the clinical and surgical outcomes among early initiators, a cohort of patients with more severe disease who traditionally had poorer outcomes.

In our cohort, all patients received standard induction regimens of infliximab or adalimumab. However, improved long-term surgical and clinical outcomes may have been achieved if an accelerated or intensified induction regimen were implemented. In a retrospective analysis of 50 patients with acute severe UC, Gibson et al. found a 33% reduction in colectomy rate (40% versus 6.7%, *p* = 0.039) with accelerated infliximab induction regimen (three doses administered within a median time of 24 days) compared to standard dosing at weeks 0, 2, and 6 [30]. Similar to our early initiator cohort, patients treated with accelerated infliximab induction had early UC (median disease duration 0.45 years) and severe disease activity (median induction CRP 67.1 mg/L, endoscopic Mayo subscore 2 to 3); nevertheless, Gibson et al. achieved colectomy rates with accelerated infliximab induction which are even lower than those reported in our cohort or the ACT trials. This suggests that early anti-TNF therapy can be effective for reducing need for surgery, but dose optimization is critical.

Both infliximab and adalimumab have been shown to reduce UC-related hospitalizations compared to placebo in RCTs [13, 16]. In CD, Mandel et al. showed that introduction of early anti-TNF therapy within three years of diagnosis reduced hospitalization rates. However, similar to our study, the authors demonstrated no benefit to earlier anti-TNF induction for reducing UC-related hospitalizations [21]. This likely reflects challenges with inducing deep sustained remission and disease severity early in the course of UC [19], but differences in disease symptomatology may also be a contributing factor. UC flares typically present dramatically with difficult-to-ignore bloody diarrhea, tenesmus, and urgency. In contrast, even uncontrolled CD-related inflammation may be asymptomatic, decreasing the likelihood of CD patients presenting to medical attention or being hospitalized [31].

Interestingly, in multivariate analysis, anti-TNF induction after 2009 was a predictor of clinical secondary loss of response and dose escalation. This may reflect changes in clinical practice patterns, particularly with respect to increased vigilance to confirm mucosal healing by endoscopy.

There are several limitations to our study. Primarily, the validity of this study may be challenged by the comparability of the early and late anti-TNF initiator cohorts. Due to nonrandomized design, the early initiator cohort included patients with more severe and active disease. As discussed previously, early versus late biologic introduction would be challenging to randomize prospectively; therefore, we specifically restricted the inclusion criteria and adjusted thoroughly for confounders to minimize selection bias. Despite these efforts, subclinical disease activity may not have been adequately captured. However, the counter argument is that our cohort more accurately reflects real-life clinical practice wherein early anti-TNF therapy is rarely introduced first line for mild UC.

Another limitation of this study was the retrospective single-centre design. Recall bias may confound the interpretation of time to diagnosis but we believe this effect is small because all patients diagnosed after 2002 had a specific date of endoscopic or histologic evaluation. For patients diagnosed prior to the introduction of the electronic medical record in 2002, date of diagnosis was confirmed by chart review but this would not have changed the patient's allocation to the late anti-TNF initiator group. Secondly, certain factors that may affect maintenance of anti-TNF response including anti-TNF drug trough and anti-drug antibody levels, body weight, and mucosal healing achieved after treatment were inconsistently available and not included. Inability to achieve mucosal healing within one year of treatment is predictive of colectomy [32], but the nonrandomized study design prevented us from extracting consistent endoscopic response data. Finally, we identified all colectomies and hospitalizations occurring in Alberta through our provincial electronic medical record, but patients travelling abroad for procedures or inpatient care would not have been captured. We suspect this represents a very minor proportion of the population and is unlikely to affect the major findings of this study.

In conclusion, patients starting early infliximab or adalimumab therapy within three years of UC diagnosis typically have more severe symptomatic, serologic, and endoscopic evidence of disease. Considering the limitations of confounders and selection bias for disease activity and treatment indication, patients receiving early anti-TNF therapy in our cohort had similar rates of colectomy, secondary loss of response, and UC-related hospitalization compared to late initiators.

## Figures and Tables

**Table 1:** Baseline patient demographics of 115 ulcerative colitis patients receiving anti-TNF therapy at the University of Alberta Inflammatory Bowel Disease Consultation and Research Clinic between 2003 and 2014.

	Early initiation of anti-TNF^**∗**^	Late initiation of anti-TNF	*p* value
*n (%)*	57 (49.6)	58 (50.4)	

*Male (%)*	33 (57.9)	32 (55.2)	0.77

*Median age (years, IQR)*			
Age at diagnosis	28.9 (22.1–44.9)	25.8 (18.9–36.4)	0.06
Age at anti-TNF induction	30.7 (22.8–45.6)	35.9 (27.0–44.5)	0.17

*Anti-TNF agent*			
Infliximab	40 (70.2)	38 (65.5)	0.59
Adalimumab	17 (29.8)	20 (34.5)	

*Median time to anti-TNF treatment after diagnosis (weeks, IQR)*	38.1 (23.3–91.0)	414.0 (254.0–561.3)	**<0.0001**

*Year of anti-TNF induction (%)*			
2003–2008	23 (40.4)	26 (44.8)	0.63
2009–2014	34 (59.6)	32 (55.2)	

*Active or former smoker (%)* ^**∗∗**^	11 (19.3)	15 (25.9)	0.76

*Montreal classification, location at anti-TNF induction (%)*			
E2, left sided distal UC	14 (24.6)	18 (31.0)	0.44
E3, extensive UC (pancolitis)	43 (75.4)	40 (69.0)	

*Disease activity* ^**∗∗∗**^			
Endoscopic Mayo score at diagnosis (mean, ±SD)	2.17 (±0.73)	1.96 (±0.61)	0.19
Endoscopic Mayo score at anti-TNF induction (mean, ±SD)	2.46 (±0.66)	1.86 (±0.67)	**<0.001**
Partial Mayo score at anti-TNF induction (mean, ±SD)	6.38 (±2.27)	5.67 (±1.95)	0.08
Median C-reactive protein (mg/L, IQR)	35.7 (9.2–80.0)	6.4 (2.0–13.6)	**<0.001**

*Previous UC treatment exposures*			
5-Aminosalicylates	53 (93.0)	55 (94.8)	0.68
Azathioprine or methotrexate	46 (80.7)	52 (89.7)	0.18

*Concurrent treatment at anti-TNF induction (%)*			
5-Aminosalicylates	30 (52.6)	32 (55.2)	0.79
Azathioprine or methotrexate	31 (54.4)	29 (50.0)	0.64
Corticosteroids	40 (70.2)	32 (55.2)	0.10

*Mean total follow-up duration (weeks, ±SD)*	153.4 (±84.3)	168.3 (±106.4)	0.41

^*∗*^Early anti-TNF initiation defined as receiving first induction dose of infliximab or adalimumab within three years of diagnosis.

^**∗∗**^Smoking status unavailable for 38 patients.

^**∗∗∗**^Endoscopic Mayo score unavailable for 37 patients at diagnosis and two patients at anti-TNF induction.

**Table 2:** Comparison between early and late initiators of anti-TNF therapy for colectomy, hospitalization, and clinical secondary loss of response requiring dose escalation during maintenance infliximab or adalimumab.

	Early initiation of anti-TNF^*∗*^	Late initiation of anti-TNF	*p* value
*Colectomy outcomes*			
Colectomy (*n*, %)	10 (17.5)	5 (8.6)	0.16
Colectomy rate (per 100 patient-years, 95% CI)	6.0 [2.9–11.0]	2.7 [0.9–6.2]	0.13
Median time to colectomy after anti-TNF (weeks, IQR)	49.1 (35.3–125.6)	119.0 (86.9–197.0)	0.61

*Indications for colectomy*			
Medically refractory disease	8 (80.0)	3 (60.0)	0.34
Disease-related complication	2 (20.0)	1 (20.0)	
Dysplasia or colorectal malignancy	0 (0)	1 (20.0)	

*Surgical urgency (%)*			
Elective surgery	5 (50.0)	4 (80.0)	0.26
Emergent surgery	5 (50.0)	1 (20.0)	

*Clinical hospitalization outcomes*			
UC-related hospitalization (*n*, %)	25 (43.9)	16 (27.6)	0.07
UC-related hospitalization rate (per 100 patient-years, 95% CI)	26.8 [19.6–35.9]	13.9 [9.1–20.4]	**0.01**
Median time to first UC-related hospitalization (weeks, IQR)	25.6 (8.6–68.7)	52.5 (28.9–96.4)	0.08

*Clinical secondary loss of response outcomes*			
Secondary loss of response requiring dose escalation (*n*, %)	28 (49.1)	34 (58.6)	0.31
Secondary loss of response rate (per 100 patient-years, 95% CI)	16.7 [11.1–24.1]	18.2 [12.6–25.4]	0.74
Median time to dose escalation (weeks, IQR)	43.6 (23.6–70.9)	48.2 (23.4–109.9)	0.80

^*∗*^Early anti-TNF initiation defined as receiving first induction dose of infliximab or adalimumab within three years of diagnosis.

**Table 3:** Summary of univariate and multivariate Cox regression models for UC-related hospitalization, colectomy, and clinical secondary loss of response requiring dose escalation during maintenance infliximab or adalimumab therapy.

	Univariate hazard ratio [95% CI]	*p* value	Multivariate hazard ratio [95% CI]	*p* value
*Hazard ratios for UC-related hospitalization* ^**∗**^				
Early anti-TNF induction within three years of diagnosis	1.83 [0.98–3.43]	0.06	1.66 [0.84–3.30]	0.15
Anti-TNF induction 2009–2014	1.86 [0.96–3.63]	0.07	1.90 [0.96–3.76]	0.07
Mayo endoscopic severity at anti-TNF induction	1.43 [0.92–2.24]	0.11	1.22 [0.77–1.94]	0.40

*Hazard ratios for colectomy* ^**∗**^				
Early anti-TNF induction within three years of diagnosis	2.56 [0.80–8.19]	0.11	2.02 [0.57–7.20]	0.28
Mayo endoscopic severity at anti-TNF induction	1.66 [0.76–3.63]	0.20	1.35 [0.58–3.14]	0.49

*Hazard ratios for clinical secondary loss of response* ^**∗**^				
Anti-TNF induction 2009–2014	1.65 [0.96–2.82]	0.07	1.95 [1.05–3.61]	0.03
C-reactive protein ≥ 8 mg/L	1.50 [0.88–2.57]	0.14	1.44 [0.78–2.64]	0.24
Mayo endoscopic severity at anti-TNF induction	0.76 [0.54–1.07]	0.12	0.74 [0.50–1.11]	0.15
Concurrent steroids at anti-TNF induction	1.52 [0.88–2.61]	0.13	1.53 [0.82–2.85]	0.19

^**∗**^In univariate analysis, choice of anti-TNF agent, gender, age, disease extent by Montreal classification, endoscopic Mayo severity score at diagnosis, partial Mayo symptom score, and concurrent use of immunomodulators or 5-ASA did not have *p* values < 0.2 and were excluded from the multivariate regression analysis.

## Additional Points

This paper was presented in the Canadian Digestive Disease Week 2016 conference in Montreal, Canada.
